# Increasing Consumption of Antibiotics during the COVID-19 Pandemic: Implications for Patient Health and Emerging Anti-Microbial Resistance

**DOI:** 10.3390/antibiotics12010045

**Published:** 2022-12-28

**Authors:** Shahana Seher Malik, Sunil Mundra

**Affiliations:** 1 Department of Biology, College of Science, United Arab Emirates University, Al Ain P.O. Box 15551, United Arab Emirates; 2 Khalifa Center for Genetic Engineering and Biotechnology, United Arab Emirates University, Al Ain P.O. Box 15551, United Arab Emirates

**Keywords:** AMR, antibiotics, cephalosporin, COVID-19, co-infection, secondary infection

## Abstract

The emergence of COVID-19 infection led to the indiscriminate use of antimicrobials without knowing their efficacy in treating the disease. The gratuitous use of antibiotics for COVID-19 treatment raises concerns about the emergence of antimicrobial resistance (AMR). In this systematic review, we performed a thorough systematic search using Preferred Reporting Items for Systematic Reviews and Meta-Analyses (PRISMA) guidelines of scientific databases (Scopus, Web of Science, and PubMed) to identify studies where antibiotics were prescribed to treat COVID-19 (December 2019 to December 2021). Of 970 identified studies, 130 were included in our analyses. Almost 78% of COVID-19 patients have been prescribed an antibiotic. Cephalosporins were the most prescribed (30.1% of patients) antibiotics, followed by azithromycin (26% of patients). Antibiotics were prescribed for COVID-19 patients regardless of reported severity; the overall rate of antibiotic use was similar when comparing patients with a severe or critical illness (77.4%) and patients with mild or moderate illness (76.8%). Secondary infections were mentioned in only 11 studies. We conclude that concerns related to COVID-19 and the lack of treatment strategy led to the overuse of antibiotics without proper clinical rationale. Based on our findings, we propose that antimicrobial stewardship should be retained as a priority while treating viral pandemics.

## 1. Introduction

During the current coronavirus disease 2019 (COVID-19) pandemic, antimicrobial use increased tremendously due to the lack of proper treatment strategies [1]. Although COVID-19 is a viral disease that is untreatable by antibiotics, viral respiratory infections can progress to bacterial pneumonia, co-infection, bacterial superinfection, and other nosocomial infections requiring antibiotic administration.

The overuse of antibiotics for treating COVID-19 patients was a result of (a) panic about an unknown disease, (b) similar symptoms to pneumonia, and (c) a higher death rate in communities with weaker immunity [2,3,4]. Moreover, the bacterial co-infection rate was almost 16%, and the use of antibiotics (especially broad-spectrum) increased to more than 72% during the current pandemic [3].

The consumption of antibiotics during COVID-19 increased tremendously, and there are various factors contributing to the spread of AMR [5,6]. Enhanced hospital exposure is silently contributing to the emerging rate of antimicrobial resistance (AMR), which causes about 700,000 deaths per year globally [7]. The widespread use of antibiotics to control pandemics might also increase resistant organisms [8,9]. The current state of antibiotic use in COVID-19 projects approximately 10 million deaths by 2050 [9,10,11,12,13]. The evidence suggests that the COVID-19 pandemic is increasing the rate of AMR through the unnecessary use of antibiotics [13]. Therefore, it is critical to strengthen antimicrobial stewardship (AMS) and formulate policies for the use of antibiotics [12].

The driving factors for the use of antibiotics include lack of proper awareness in the public, potential access and affordability to antibiotics without prescription, and use of leftover antibiotics from earlier prescriptions. While some other driving factors are insufficient training during the early phase of clinical practice, irrational prescriptions to promote a pharmaceutical company, and inadequate diagnostic process [14].

The lack of new antibiotics development for the past three decades, termed as “discovery void”, is due to the scarcity of research to find new antimicrobials [12,14]. COVID-19 has also disrupted the production, delivery, and processing of antimicrobials. During the current pandemic, the demand to find a treatment has led to a shift in research resources and funds to new antivirals and vaccines instead of antibiotics [15]. Furthermore, the shortage and rerouting of medical funds during COVID-19 have affected many small labs that produce medicines and vaccines for local markets. This deficiency of narrow-spectrum antibiotics can cause an increase in AMR [16]. Clinical trials were also disrupted as the hospitals focused on COVID-19 [17]. Further, there is a need to start clinical trials of many antimicrobials. The results of these trials are expected to improve COVID-19 treatments and patient outcomes. 

The studies focusing on AMR have raised concern about the inflammatory effects of administered drugs [18]. Therefore, different countries have formulated many guidelines for antimicrobial use during the pandemic. However, the World Health Organization (WHO) has recommended avoiding antibiotic use for patients with mild to moderate symptoms of bacterial or COVID-19 infections [19]. According to WHO, for severe cases, only low-potency antibiotics are recommended, and in the case of aged persons, the antibiotics included in the access list of WHO (https://aware.essentialmeds.org/groups (accessed on 25 August 2021)) can only be prescribed [20]. Nonetheless, these guidelines are insufficient to limit AMR emergence. There is a strict need for evidence-based guidelines for AMS during and/or post-COVID-19 pandemic. There is a need to analyze the trends for the pandemic’s spread and the complete details of antibiotics used globally since the COVID-19 outbreak.

Here, we performed a systematic analysis to assess changes in antibiotic use during the COVID-19 era and how these changes might impact AMR. 

## 2. Results

**Figure 1:**
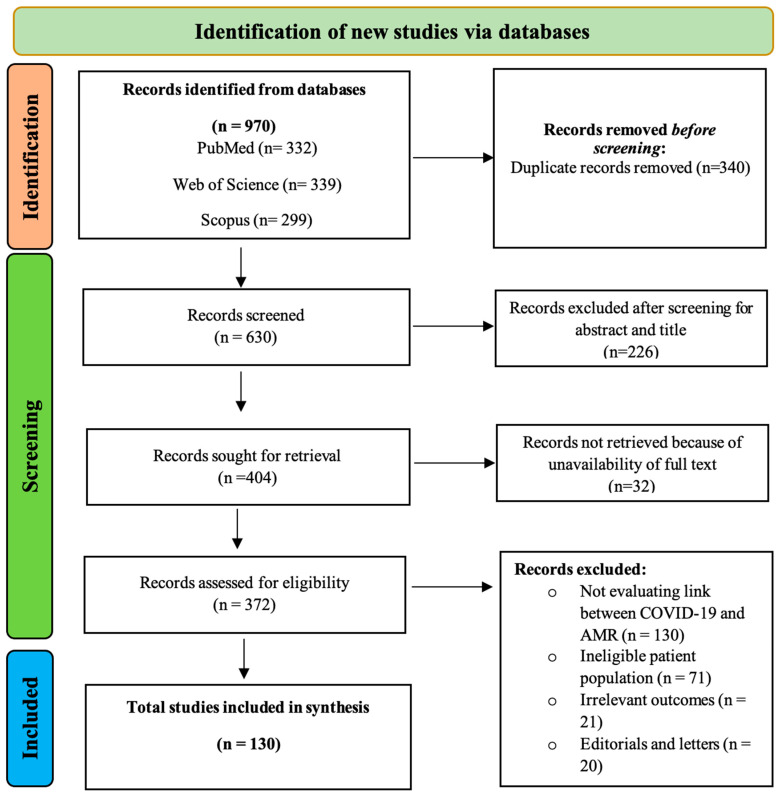
PRISMA workflow for the literature identification from 3 databases (PubMed, Web of Science, and Scopus), screening, and final selection of research studies for synthesis study. The exclusion criteria of the workflow include duplicate studies from multiple databases, screening based on the irrelevance of title and abstract, removal of records due to unavailability of full-text studies, studies not evaluating the link between COVID-19 and AMR, and having irrelevant outcomes, editorials, and letters.

Based on our keyword search using PRISMA, we selected 970 research articles. Of these, 260 articles (84 review studies, 172 journal articles, and 4 short surveys) were further screened based on the exclusion and inclusion criteria. We further excluded another 130 studies that did not report the application of antimicrobials while treating COVID-19 patients. Finally, a total of 130 articles were selected for final systematic synthesis (Figure 1, Table S1). Among selected studies, most were conducted in the USA, followed by the UK, India, Italy, and China.

### 2.1. Usage of Antibiotics according to the Severity of Illness

**Figure 2:**
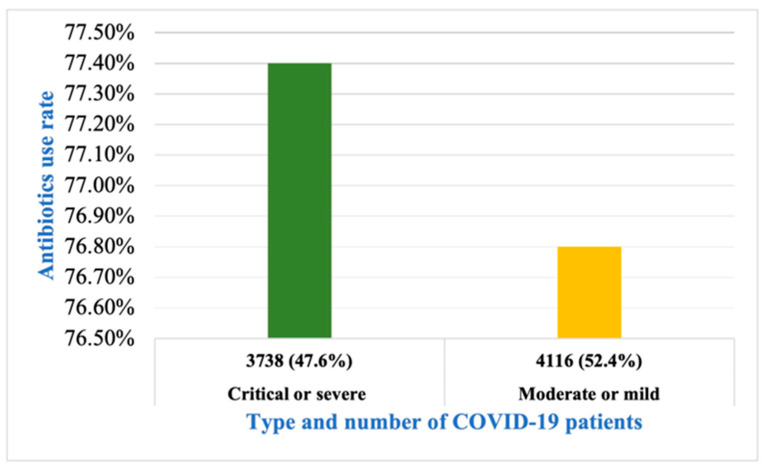
Rate of antibiotic use in COVID-19 patients according to the severity of illness.

Overall, 47.6% of patients were suffering from severe or critical illness, while the remaining cases were of mild or moderate nature. Almost 78% of patients were prescribed antibiotics. A minor difference was seen in the prescription of antibiotics among severe or critical and moderate or mild patients (77.4% and 76.8%; Figure 2).

### 2.2. Use of Antibiotics and Related Health Effects

**Figure 3:**
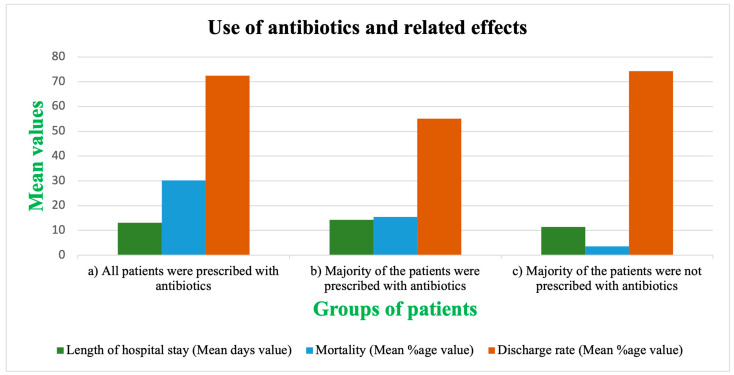
The use of antibiotics in COVID-19 patients divided into three groups (**a**) all patients using antibiotics, (**b**) the majority of the patients using antibiotics, and (**c**) the majority of the patients not using antibiotics) and related effects (length of hospital stay, mortality, and discharge rate).

The mortality rate was higher in cases where all patients used antibiotics than in cases in which most patients were not given antibiotics. Length of hospital stay (LOS) was higher in the patients’ group, where not all, but the majority, were given antibiotics. The discharge rate was highest amongst those patients who were not given antibiotics compared to the group where most of the patients received antibiotics (Figure 3).

### 2.3. Bacterial Co-Infections and Resulting Health Outputs

Of the total, 11 studies reported secondary infections in COVID-19 patients. In our analysis, 51.3% of the patients showed secondary infections, and 57.5% were in critical condition (Table 1).

**Figure 4:**
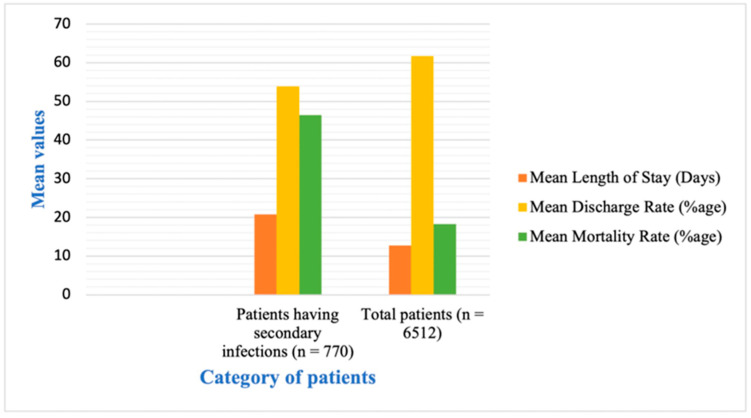
Comparison of hospital stay, discharge rate, and mortality rate in COVID-19 patients with and without secondary infections.

Among all patients, the percentage of confirmed co-infections was 12%. The patients with secondary infections also had a higher mean value of LOS and mortality rate. At the same time, the hospital discharge rate was lower in these patients (Figure 4).

### 2.4. Antimicrobials’ Usage Trends

**Figure 5:**
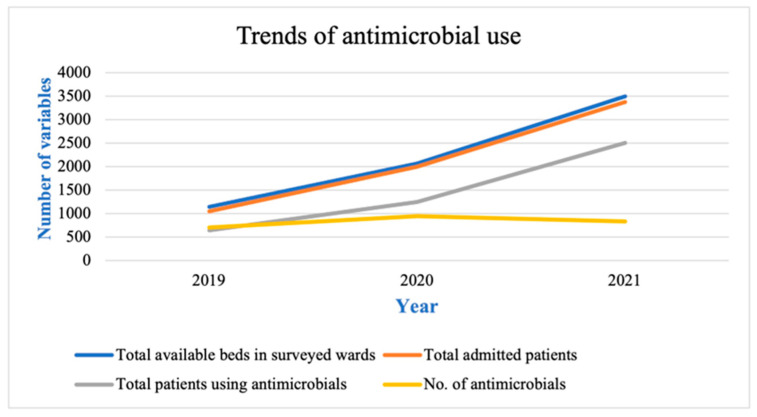
Trends of antimicrobial use in hospitalized COVID-19 patients along with total available beds in surveyed hospital wards.

The number of antimicrobial types prescribed showed a very slight increase in 2020 and 2021 compared to 2019, but the number of patients using antimicrobials increased tremendously over that time. In 2019 the number of different antimicrobials being used was 645, and in 2021 it increased by almost 4-fold to 2503. The total number of admitted patients and available beds in the surveyed wards also increased simultaneously from the year 2019 to the year 2021 as the number of COVID-19 cases surged (Figure 5).

## 3. Discussion

The extensive use of antibiotics has increased since the COVID-19 outbreak, increasing concerns about AMR. This study includes only the data of hospitalized patients because very little data are available about those confined to their homes with mild or moderate symptoms. This is probably because most research studies are performed on patients reporting to medical facilities.

We found that >78% of COVID-19 patients were recommended to use antibiotics (Figure 2). The broad-spectrum antibiotic azithromycin was the most frequently prescribed, followed by ceftriaxone, moxifloxacin, meropenem, and tazobactam. The excessive use of broad-spectrum antibiotics without proper clinical justifications by healthcare persons has raised concerns about AMR amplification [21].

It has been previously reported that azithromycin was the most common antimicrobial agent used while treating COVID-19 [22]. Azithromycin is very efficient in treating pneumonia, but there is no proof of its effect on viruses. There are studies that clearly show that excessive use of azithromycin can result in antimicrobial resistance, which indicates that overuse or misuse of this antibiotic during COVID-19 can contribute to AMR [23,24]. The WHO also reported the excess use of azithromycin for COVID-19 treatment even without approval [25]. It is classified as a critically important antimicrobial used in humans [26]. Persistent use of this antimicrobial can result in AMR, causing a serious threat to survival in severe infection cases. Third-generation cephalosporins such as ceftriaxone have long been mostly used in intensive care units (ICUs). As the consumption of other antibiotics changed during the pandemic, cephalosporins were also being repurposed [27]. Karami et al. provided clear evidence of cephalosporin use in more than 200 of 556 cases [28].

Doxycycline is another drug that could help treat COVID-19, which is used because of its antiviral and anti-inflammatory properties [29]. Many broad-spectrum antibiotics, such as fluoroquinolones and cephalosporins, were reportedly used in more than 74% of patients. If this use of broad-spectrum antibiotics is not stopped, it could lead to AMR. This could lead to very few remaining options for treating infections, and these options could become unaffordable for underdeveloped countries.

Increasing telehealth services also caused excessive antibiotic use due to the unavailability of the proper diagnostic channel and determining the nature of the illness [30]. The best treatment options for COVID-19 were unknown, so experimental treatments played a large role in inappropriate drug prescriptions during this pandemic period [17]. Due to this vagueness in the COVID-19 era, healthcare workers prescribed antibiotics based on the assumption that the drug’s potential threat would be negligible compared to its benefit [31]. Previous studies also mentioned increased antimicrobial use due to self-medication [32,33,34]. This was more common in areas where antimicrobials could be easily accessed without any prescription [16,35]. One study reported that to avoid visits to any healthcare facility, about 20% of Iranians used self-prescribed medicines for their sickness [36]. 

We found that the recommendation rate of antibiotics does not change with the severity of the disease (Figure 2). Both mild or moderate and severe or critical illness patient groups were given antibiotics, although the severe cases had a greater chance of suffering from a secondary infection. The high rate of antibiotic application in mild cases is also very alarming. The results show that the mortality rate was higher in cases where all patients used antibiotics than in cases in which most patients were not given antibiotics. Length of hospital stay (LOS) was higher in the patients’ group, where not all, but the majority, were given antibiotics (Figure 3). In our analysis, 51.3% of the patients showed secondary infections, and 57.5% were in critical condition (Table 1). We also found that the co-infection rate was higher in patients with severe COVID-19 symptoms, and the mortality rate was greater in patients with some co-infection or secondary infection (Figure 4). Although the rate of antibiotic prescription is enormous, secondary infections were only reported in 14.3% of cases, along with 3.5% of reported cases of co-infections [20]. The bacterial co-infection of COVID-19 patients has been reported in many studies worldwide [37,38,39,40,41]. The co-infection rate was almost 28% in Europe [42]. Patients with mild or medium symptoms were not reported for co-infection because these patients were not checked or tested for infection [43,44,45]. Moreover, in many cases, when the specimen was taken, the co-infections were reported to be more associated with hospital-acquired than community-acquired infections. 

Our analysis shows that the number of antimicrobial types increased only slightly in 2020 and 2021 compared to 2019, but the number of patients using antimicrobials increased tremendously (a four-fold increase) (Figure 5). The total number of admitted patients also increased from 2019 to 2021. This indicates a positive relationship between the number of COVID-19 patients and the antimicrobials consumption, contributing to AMR. A higher rate of AMR could be predicted in low- and middle-income countries because of a lack of awareness and stewardship programs, poor lab facilities, and a lack of proper rules for accessing antibiotics without prescription [46]. COVID-19 can be more easily spread to areas that are more populated and lack proper hygiene facilities. In low- and middle-income countries, allocating resources to COVID-19 is very difficult as their healthcare facilities already lack proper funds, which is an additional burden to their healthcare systems [32].

In many studies, classes of antibiotics are mentioned, but information related to the use of antibiotics according to disease severity was not available. The variation in the number of research studies across regions might have affected our results, as there is a huge difference in the number of patients and local regulations for COVID-19 [47]. Moreover, the selection biases (not all the studies included in this systematic review are directly discussing AMR and COVID-19) are there. In this paper, we mostly focused on selecting the papers that provide evidence of antibiotic use during COVID-19, as the data related to antibiotics use can help in providing a clear understanding of the rate of AMR prevalence at this time. Healthcare professionals were also under pressure during the COVID-19 pandemic. In small hospitals where healthcare facilities are insufficient, they must try every possible option to save the life of patients. The diagnosis of secondary infections is very costly, and most hospitals lack this facility, leading doctors to prescribe antibiotics even when they are not needed. This needs to be managed as the excessive use of antimicrobials raises the threat of AMR.

### Antimicrobial Stewardship (AMS)

Our analysis demonstrates that COVID-19 has crucial implications for AMR. The overuse or misuse of antibiotics to control COVID-19 symptoms, even without any co-infection, has worsened the situation. The world health organization has clear guidelines in this regard; patients with mild symptoms should not be prescribed antibiotics. The antibiotics should only be prescribed when there is clear evidence of bacterial co-infection. Better diagnostics are required to identify patients with secondary infections to avoid misuse of antimicrobials. AMS programs can assist in properly using antimicrobials by reviewing every prescribed medication. The data to date show that the use of antimicrobials is much higher than needed. For real-time data review and dissemination, additional efforts are needed to improve AMS. These efforts will not only help control COVID-19 but will have a major role in controlling the future pandemic of AMR.

## 4. Materials and Methods

The study was conducted to identify and analyze the research studies reporting the use of antimicrobials (especially azithromycin, doxycycline, clarithromycin, ceftriaxone, erythromycin, amoxicillin, amoxicillin-clavulanic acid, ampicillin, gentamicin, benzylpenicillin) for treating COVID-19. The data were reported using Preferred Reporting Items for Systematic Reviews and Meta-Analyses (PRISMA) guidelines for systematic review, and its protocol is registered in the PROSPERO register [48,49]. In order to maximize the authenticity of our findings, we tried to include as many research studies as possible in this analysis. All data were from hospitalized COVID-19 patients, and the final selection was based on RT-PCR-confirmed COVID-19 cases. 

### 4.1. Strategy for Data Search

The study included all papers from several scientific databases such as Scopus, Web of Science, and PubMed-associated peer-reviewed journals published since the COVID outbreak (December 2019 till December 2021). The keywords and search terms used were “Antimicrobial resistance” and “coronavirus” or “COVID-19” and “Antimicrobial resistance” and “Antimicrobial stewardship” or “Antibiotic resistance” and “COVID-19”.

### 4.2. Enclosure and Elimination Criteria for Research Studies

We included all the studies: (1) related to COVID-19 patients from communities and hospitals; (2) where antibiotics were prescribed during treatment; and (3) published in English (or in press). We selected cohort studies, cross-sectional studies, case-control studies, randomized control studies, and descriptive and observational research studies related to the use of antimicrobials in COVID-19 patients. We included those studies reporting the patients and use of antibiotics without any discrimination in gender, age, color, country, or community. We also considered the studies that reported antibiotics usage without specifying their types or treatment outcomes. We excluded those research studies that overlapped (duplicate data), contained unreliable data (short reports containing no proper results), were published in the form of editorials and notes, and studies related to engineering and earth sciences. Our analysis did not consider the studies related to animal experimentation, molecular mechanism, drug modeling, and other aspects of COVID-19, except for the use of antibiotics/antimicrobials.

### 4.3. Data Extraction

After cross-checking for the study accuracy and duplicates, data extraction was carried out based on the year of study, type of article, design or idea of the study, area/country of study, the sternness of COVID-19, rate of bacterial co-infections (infection acquired with first 24 to 48 h of hospitalization) and secondary infections (infection acquired after 24 to 48 h of hospitalization), prescribed antimicrobials, and the number of patients consuming those antimicrobials. The extraction of data from selected research studies comprised of details of publication, region of study, number of reported patients, type of study (e.g., case study, cohort study, descriptive study), condition of patients (mild, severe, or critical), rate of antimicrobial usage and recommendation, the scenarios of prescription, time of hospital stay, and mortality rate. More than half of the studies reported disease severity as mild, moderate, critical, and severe, while the others used just mild, moderate, and severe. We categorized disease severity into two major groups: mild or moderate and severe or critical.

### 4.4. Data Synthesis and Analysis

We analyzed all the selected studies where details about medicine prescriptions were available. We also investigated antimicrobials and antibiotics use according to the severity of illness, types that were used most often to treat COVID-19, health effects related to the use of antibiotics, bacterial co-infections, and related outputs, and trends of antimicrobial usage at the time of COVID-19. We also explored the length of hospital stay (LOS), rate of discharge, and mortality (patients still in the hospital at the time of publication were excluded from this calculation). 

## 5. Conclusions

The study reveals that most antibiotics (mainly azithromycin and cephalosporins) prescribed during COVID-19 treatment were not related to disease severity since antibiotics were prescribed to patients with mild symptoms. Moreover, antibiotics were recommended without any medical or biological indication of secondary bacterial infection and thus may not have been beneficial in remedying COVID-19 infection. The extensive use of antibiotics might augment antibiotic resistance, thereby eroding the usefulness of currently available antibiotics. The overuse of antibiotics may silently trigger future pandemics due to the emerging AMR [50].

## Figures and Tables

**Table 1:** The comparison of disease severity, mean length of stay in hospital, mean discharge rate, and mean mortality rate among patients with secondary infections and normal COVID-19 patients.

Serial No.	Category	Critical or Severe n (%age)	Moderate or Mild *n* (%)	Mean Length of Stay (Days)	Mean Discharge Rate (%age)	Mean Mortality Rate (%age)
**1**	Patients with secondary infections (*n* = 770)	443 (57.5%)	327 (42.5%)	20.8	53.9	46.5
**2**	Total patients (*n* = 6512)	2807 (43.2%)	3705 (56.8%)	12.7	61.7	18.3

## Data Availability

Not applicable.

## Supplementary Materials

The following supporting information can be downloaded at: https://www.mdpi.com/article/10.3390/antibiotics12010045/s1, Table S1: List of studies included for final Systematic Review.

## References

[1] Baby B., Devan A.R., Nair B., Nath L.R. (2021). The Impetus of COVID-19 in Multiple Organ Affliction Apart from Respiratory Infection: Pathogenesis, Diagnostic Measures and Current Treatment Strategy. Infect. Disord.-Drug Targets Former. Curr. Drug Targets-Infect. Disord..

[2] Stankovska G., Memedi I., Dimitrovski D. (2020). Coronavirus COVID-19 disease, mental health and psychosocial support. Soc. Regist..

[3] Townsend L., Hughes G., Kerr C., Kelly M., O’Connor R., Sweeney E., Doyle C., O’Riordan R., Martin-Loeches I., Bergin C. (2020). Bacterial pneumonia coinfection and antimicrobial therapy duration in SARS-CoV-2 (COVID-19) infection. JAC-Antimicrobial Resist..

[4] Wang L., Alexander C.A. (2020). COVID-19 Compared with Other Viral Diseases: Novelties, Progress, and Challenges. Electron. J. Gen. Med..

[5] Mazumder P., Kalamdhad A., Chaminda G.T., Kumar M. (2021). Coalescence of co-infection and antimicrobial resistance with SARS-CoV-2 infection: The blues of post-COVID-19 world. Case Stud. Chem. Environ. Eng..

[6] Rasul C. (2021). Indiscriminate use of antimicrobials during COVID-19 pandemic. Bangladesh Med. J. Khulna.

[7] Machowska A., Lundborg C.S. (2018). Drivers of Irrational Use of Antibiotics in Europe. Int. J. Environ. Res. Public Health.

[8] Caselli E. (2017). Hygiene: Microbial strategies to reduce pathogens and drug resistance in clinical settings. Microb. Biotechnol..

[9] Hashmi F.K., Atif N., Malik U.R., Saleem F., Riboua Z., Hassali M.A., Butt M.H., Mallhi T.H., Khan Y.H. (2020). In Pursuit of COVID-19 Treatment Strategies: Are We Triggering Antimicrobial Resistance?. Disaster Med. Public Health Prep..

[10] Cong W., Poudel A., Alhusein N., Wang H., Yao G., Lambert H. (2021). Antimicrobial Use in COVID-19 Patients in the First Phase of the SARS-CoV-2 Pandemic: A Scoping Review. Antibiotics.

[11] Baggs J., Rose A.N., McCarthy N.L., Wolford H., Srinivasan A., A Jernigan J., Reddy S.C. (2022). Antibiotic-Resistant Infections Among Inpatients with Coronavirus Disease 2019 (COVID-19) in US Hospitals. Clin. Infect. Dis..

[12] Getahun H., Smith I., Trivedi K., Paulin S., Balkhy H.H. (2020). Tackling antimicrobial resistance in the COVID-19 pandemic. Bull. World Health Organ..

[13] A Strathdee S., Davies S.C., Marcelin J.R. (2020). Confronting antimicrobial resistance beyond the COVID-19 pandemic and the 2020 US election. Lancet.

[14] Zawahir S., Le H., Nguyen T.A., Beardsley J., Duc A.D., Bernays S., Viney K., Hung T.C., McKinn S., Tran H.H. (2021). Standardised patient study to assess tuberculosis case detection within the private pharmacy sector in Vietnam. BMJ Glob. Health.

[15] Ukuhor H.O. (2020). The interrelationships between antimicrobial resistance, COVID-19, past, and future pandemics. J. Infect. Public Health.

[16] Khor W.P., Olaoye O., D’Arcy N., Krockow E.M., Elshenawy R.A., Rutter V., Ashiru-Oredope D. (2020). The Need for Ongoing Antimicrobial Stewardship during the COVID-19 Pandemic and Actionable Recommendations. Antibiotics.

[17] Nieuwlaat R., Mbuagbaw L., Mertz D., Burrows L.L., E Bowdish D.M., Moja L., Wright G.D., Schünemann H.J. (2020). Coronavirus Disease 2019 and Antimicrobial Resistance: Parallel and Interacting Health Emergencies. Clin. Infect. Dis..

[18] Buetti N., Mazzuchelli T., Priore E.L., Balmelli C., Llamas M., Pallanza M., Elzi L., Consonni V., Trimboli P., Forni-Ogna V. (2020). Early administered antibiotics do not impact mortality in critically ill patients with COVID-19. J. Infect..

[19] World Health Organization (2021). Living Guidance for Clinical Management of COVID-19: Living Guidance.

[20] WHO (2020). Responding Community Spread COVID-19 Ref WHO COVID-19 Community Transmission 2020–2021.

[21] Tenforde M.W., Kim S.S., Lindsell C.J., Rose E.B., Shapiro N.I., Files D.C., Gibbs K.W., Erickson H.L., Steingrub J.S., Smithline H.A. (2020). Symptom Duration and Risk Factors for Delayed Return to Usual Health among Outpatients with COVID-19 in a Multistate Health Care Systems Network—United States, March–June 2020. MMWR. Morb. Mortal. Wkly. Rep..

[22] Cangini A., Fortinguerra F., Di Filippo A., Pierantozzi A., Da Cas R., Villa F., Trotta F., Moro M.L., Gagliotti C. (2020). Monitoring the community use of antibiotics in Italy within the National Action Plan on antimicrobial resistance. Br. J. Clin. Pharmacol..

[23] Hooda Y., Tanmoy A.M., Sajib M.S.I., Saha S. (2020). Mass azithromycin administration: Considerations in an increasingly resistant world. BMJ Glob. Health.

[24] Mack I., Sharland M., A Berkley J., Klein N., Malhotra-Kumar S., Bielicki J. (2019). Antimicrobial Resistance Following Azithromycin Mass Drug Administration: Potential Surveillance Strategies to Assess Public Health Impact. Clin. Infect. Dis..

[25] E Lane J.C., Weaver J., Kostka K., Duarte-Salles T., Abrahao M.T.F., Alghoul H., Alser O., Alshammari T.M., Biedermann P., Banda J.M. (2020). Risk of hydroxychloroquine alone and in combination with azithromycin in the treatment of rheumatoid arthritis: A multinational, retrospective study. Lancet Rheumatol..

[26] World Health Organization (2019). Critically Important Antimicrobials for Human Medicine.

[27] Durojaiye A.B., Clarke J.-R.D., Stamatiades G.A., Wang C. (2020). Repurposing cefuroxime for treatment of COVID-19: A scoping review of in silico studies. J. Biomol. Struct. Dyn..

[28] Karami Z., Knoop B.T., Dofferhoff A.S.M., Blaauw M.J.T., Janssen N.A., van Apeldoorn M., Kerckhoffs A.P.M., van de Maat J.S., Hoogerwerf J.J., Oever J.T. (2020). Few bacterial co-infections but frequent empiric antibiotic use in the early phase of hospitalized patients with COVID-19: Results from a multicentre retrospective cohort study in The Netherlands. Infect. Dis..

[29] Kearns F.L., Sandoval D.R., Casalino L., Clausen T.M., Rosenfeld M.A., Spliid C.B., Amaro R.E., Esko J.D. (2022). Spike-heparan sulfate interactions in SARS-CoV-2 infection. Curr. Opin. Struct. Biol..

[30] Rawson T.M., Ming D., Ahmad R., Moore L.S.P., Holmes A.H. (2020). Antimicrobial use, drug-resistant infections and COVID-19. Nat. Rev. Microbiol..

[31] Hsu J. (2020). How COVID-19 is accelerating the threat of antimicrobial resistance. BMJ.

[32] Makowska M., Boguszewski R., Nowakowski M., Podkowińska M. (2020). Self-Medication-Related Behaviors and Poland’s COVID-19 Lockdown. Int. J. Environ. Res. Public Health.

[33] Tekeba A., Ayele Y., Negash B., Gashaw T. (2021). Extent of and Factors Associated with Self-Medication among Clients Visiting Community Pharmacies in the Era of COVID-19: Does It Relieve the Possible Impact of the Pandemic on the Health-Care System?. Risk Manag. Health Policy.

[34] Zhang A., Hobman E., De Barro P., Young A., Carter D., Byrne M. (2021). Self-Medication with Antibiotics for Protection against COVID-19: The Role of Psychological Distress, Knowledge of, and Experiences with Antibiotics. Antibiotics.

[35] Usman M., Farooq M., Hanna K. (2020). Environmental side effects of the injudicious use of antimicrobials in the era of COVID-19. Sci. Total. Environ..

[36] Heydargoy M.H. (2020). The Effect of the Prevalence of COVID-19 on Arbitrary Use of Antibiotics. Iran. J. Med. Microbiol..

[37] Garcia-Vidal C., Sanjuan G., Moreno-García E., Puerta-Alcalde P., Garcia-Pouton N., Chumbita M., Fernandez-Pittol M., Pitart C., Inciarte A., Bodro M. (2020). Incidence of co-infections and superinfections in hospitalized patients with COVID-19: A retrospective cohort study. Clin. Microbiol. Infect..

[38] Hughes S., Troise O., Donaldson H., Mughal N., Moore L.S.P. (2020). Bacterial and fungal coinfection among hospitalized patients with COVID-19: A retrospective cohort study in a UK secondary-care setting. Clin. Microbiol. Infect..

[39] Iacobucci G. (2020). COVID-19: Risk of death more than doubled in people who also had flu, English data show. BMJ.

[40] Lv Z., Cheng S., Le J., Huang J., Feng L., Zhang B., Li Y. (2020). Clinical characteristics and co-infections of 354 hospitalized patients with COVID-19 in Wuhan, China: A retrospective cohort study. Microbes Infect..

[41] Sharifipour E., Shams S., Esmkhani M., Khodadadi J., Fotouhi-Ardakani R., Koohpaei A., Doosti Z., Golzari S.E. (2020). Evaluation of bacterial co-infections of the respiratory tract in COVID-19 patients admitted to ICU. BMC Infect. Dis..

[42] Contou D., Claudinon A., Pajot O., Micaëlo M., Flandre P.L., Dubert M., Cally R., Logre E., Fraissé M., Mentec H. (2020). Bacterial and viral co-infections in patients with severe SARS-CoV-2 pneumonia admitted to a French ICU. Ann. Intensiv. Care.

[43] Abdoli A. (2020). Helminths and COVID-19 Co-Infections: A Neglected Critical Challenge. ACS Pharmacol. Transl. Sci..

[44] Chen X., Liao B., Cheng L., Peng X., Xu X., Li Y., Hu T., Li J., Zhou X., Ren B. (2020). The microbial coinfection in COVID-19. Appl. Microbiol. Biotechnol..

[45] Langford B.J., So M., Raybardhan S., Leung V., Westwood D., MacFadden D.R., Soucy J.-P.R., Daneman N. (2020). Bacterial co-infection and secondary infection in patients with COVID-19: A living rapid review and meta-analysis. Clin. Microbiol. Infect..

[46] Iskandar K., Molinier L., Hallit S., Sartelli M., Hardcastle T.C., Haque M., Lugova H., Dhingra S., Sharma P., Islam S. (2021). Surveillance of antimicrobial resistance in low- and middle-income countries: A scattered picture. Antimicrob. Resist. Infect. Control..

[47] Huttner B.D., Catho G., Pano-Pardo J.R., Pulcini C., Schouten J. (2020). COVID-19: Don’t neglect antimicrobial stewardship principles. Clin. Microbiol. Infect..

[48] Cohen J.F., Deeks J.J., Hooft L., Salameh J.-P., A Korevaar D., Gatsonis C., Hopewell S., A Hunt H., Hyde C.J., Leeflang M.M. (2021). Preferred reporting items for journal and conference abstracts of systematic reviews and meta-analyses of diagnostic test accuracy studies (PRISMA-DTA for Abstracts): Checklist, explanation, and elaboration. BMJ.

[49] Tricco A.C., Lillie E., Zarin W., O’Brien K.K., Colquhoun H., Levac D., Moher D., Peters M.D.J., Horsley T., Weeks L. (2018). PRISMA Extension for Scoping Reviews (PRISMA-ScR): Checklist and Explanation. Ann. Intern. Med..

[50] Ansari S., Hays J.P., Kemp A., Okechukwu R., Murugaiyan J., Ekwanzala M.D., Alvarez M.J.R., Paul-Satyaseela M., Iwu C.D., Balleste-Delpierre C. (2021). The potential impact of the COVID-19 pandemic on global antimicrobial and biocide resistance: An AMR Insights global perspective. JAC-Antimicrobial Resist..

