# Supplementary material for: Increasing Consumption of Antibiotics during the COVID-19 Pandemic: Implications for Patient Health and Emerging Anti-Microbial Resistance
Source: Antibiotics (Basel). 2022 Dec 28;12(1):45. doi: 10.3390/antibiotics12010045 (PMC9855050; doi:10.3390/antibiotics12010045)
Supplement: Supplementary file 1 [file antibiotics-12-00045-s001.zip › antibiotics-2084199-supplementary.pdf]

Manuscript ID: antibiotics-2084199
MDPI Antibiotics Journal

Title: Increasing consumption of antibiotics during the COVID-19 pandemic: Implications for patient health and emerging antimicrobial resistance

Shahana Seher Malik 1 and Sunil Mundra [1,2,*]

[1]Department of Biology, College of Science, United Arab Emirates University, Al Ain, 15551, United Arab Emirates

[2]Khalifa Center for Genetic Engineering and Biotechnology, United Arab Emirates University, Al Ain, 15551, United Arab Emirates

*Correspondence: sunilmundra@uaeu.ac.ae; Tel.: +971-7136341

Table S1: List of studies included for final Systematic Review.

1	(Saadh et al. 2021)	Saadh, Mohamed J., Abeer M. Kharshid, Heba K. Aladailah, Shahad Abunawas, and Wael Abu Dayyih. 2021. "Antibiotic Stewardship in ICU during Covid-19." *Pharmacologyonline*, 2032–39.
2	(Mahadi 2021)	Mahadi, Ashrafur Rahaman. 2021. "Post COVID Antimicrobial Resistance Threat in Lower-and Middle-Income Countries: Bangladesh." *Frontiers in Public Health* 9.
3	(Ma, Kung, and Chen 2021)	Ma, Edmond SK, K. H. Kung, and Hong Chen. 2021. "Combating Antimicrobial Resistance during the COVID-19 Pandemic." *Hong Kong Medical Journal* 27 (6): 396.
4	(Posteraro et al. 2021)	Posteraro, Brunella, Venere Cortazzo, Flora Marzia Liotti, Giulia Menchinelli, Chiara Ippoliti, Giulia De Angelis, Marilena La Sorda, Gennaro Capalbo, Joel Vargas, and Massimo Antonelli. 2021. "Diagnosis and Treatment of Bacterial Pneumonia in Critically Ill Patients with COVID-19 Using a Multiplex PCR Assay: A Large Italian Hospital's Five-Month Experience." *Microbiology Spectrum* 9 (3): e00695-21.
5	(Kalam et al. 2021)	Kalam, Abul, Shahanaj Shano, Mohammad Asif Khan, Ariful Islam, Narelle Warren, Mohammad Mahmudul Hassan, and Mark Davis. 2021. "Understanding the Social Drivers of Antibiotic Use during COVID-19 in Bangladesh: Implications for Reduction of Antimicrobial Resistance." *PloS One* 16 (12): e0261368.
6	(Jampani and Chandy 2021)	Jampani, Mahesh, and Sujith J. Chandy. 2021. "Increased Antimicrobial Use during COVID-19: The Risk of Advancing the Threat of Antimicrobial Resistance." *Health Science Reports* 4 (4).
7	(Pierce and Stevens 2021)	Pierce, Jacob, and Michael P. Stevens. 2021. "COVID-19 and Antimicrobial Stewardship: Lessons Learned, Best Practices, and Future Implications." *International Journal of Infectious Diseases* 113: 103–8.
8	(Osman et al. 2021)	Osman, Marwan, Dalal Kasir, Issmat I. Kassem, and Monzer Hamze. 2021. "Shortage of Appropriate Diagnostics for Antimicrobial

		Resistance in Lebanese Clinical Settings: A Crisis Amplified by COVID-19 and Economic Collapse." *Journal of Global Antimicrobial Resistance* 27: 72.
9	(Karataş et al. 2021)	Karataş, Mustafa, Melike Yaşar-Duman, Alper Tünger, Feriha Çilli, Şöhret Aydemir, and Volkan Özenci. 2021. "Secondary Bacterial Infections and Antimicrobial Resistance in COVID-19: Comparative Evaluation of Pre-Pandemic and Pandemic-Era, a Retrospective Single Center Study." *Annals of Clinical Microbiology and Antimicrobials* 20 (1): 1–8.
10	(Al-Hadidi et al. 2021)	Al-Hadidi, Sara H., Hashim Alhussain, Hamad Abdel Hadi, Alreem Johar, Hadi M. Yassine, Asmaa A. Al Thani, and Nahla O. Eltai. 2021. "The Spectrum of Antibiotic Prescribing during COVID-19 Pandemic: A Systematic Literature Review." *Microbial Drug Resistance* 27 (12): 1705–25.
11	(Fontana et al. 2021)	Fontana, Carla, Marco Favaro, Silvia Minelli, Maria Cristina Bossa, and Anna Altieri. 2021. "Co-Infections Observed in SARS-CoV-2 Positive Patients Using a Rapid Diagnostic Test." *Scientific Reports* 11 (1): 1–10.
12	(Adebisi, Jimoh, et al. 2021)	Adebisi, Yusuff Adebayo, Nafisat Dasola Jimoh, Isaac Olushola Ogunkola, Theogene Uwizeyimana, Alaka Hassan Olayemi, Nelson Ashinedu Ukor, and Don Eliseo Lucero-Prisno. 2021. "The Use of Antibiotics in COVID-19 Management: A Rapid Review of National Treatment Guidelines in 10 African Countries." *Tropical Medicine and Health* 49 (1): 1–5.
13	(Pelfrene, Botgros, and Cavaleri 2021)	Pelfrene, Eric, Radu Botgros, and Marco Cavaleri. 2021. "Antimicrobial Multidrug Resistance in the Era of COVID-19: A Forgotten Plight?" *Antimicrobial Resistance & Infection Control* 10 (1): 1–6.
14	(Z. Chen et al. 2021)	Chen, Zhongli, Jinsong Guo, Yanxue Jiang, and Ying Shao. 2021. "High Concentration and High Dose of Disinfectants and Antibiotics Used during the COVID-19 Pandemic Threaten Human Health." *Environmental Sciences Europe* 33 (1): 1–4.
15	(Rizk et al. 2021)	Rizk, Nesrine A., Rima Moghnieh, Nisrine Haddad, Marie-Claire Rebeiz, Rony M. Zeenny, Joya-Rita Hindy, Gabriella Orlando, and Souha S. Kanj. 2021. "Challenges to Antimicrobial Stewardship in the Countries of the Arab League: Concerns of Worsening Resistance during the COVID-19 Pandemic and Proposed Solutions." *Antibiotics* 10 (11): 1320.
16	(Tomczyk et al. 2021)	Tomczyk, Sara, Angelina Taylor, Allison Brown, Marlieke EA De Kraker, Aiman El-Saed, Majid Alshamrani, Rene S. Hendriksen, Megan Jacob, Sonja Löfmark, and Olga Perovic. 2021. "Impact of the COVID-19 Pandemic on the Surveillance, Prevention and Control of Antimicrobial Resistance: A Global Survey." *Journal of Antimicrobial Chemotherapy* 76 (11): 3045–58.
17	(Hirabayashi et al. 2021)	Hirabayashi, Aki, Toshiki Kajihara, Koji Yahara, Keigo Shibayama, and Motoyuki Sugai. 2021. "Impact of the COVID-19 Pandemic on

		the Surveillance of Antimicrobial Resistance.” *Journal of Hospital Infection* 117: 147–56.
18	(Segala et al. 2021)	Segala, Francesco Vladimiro, Davide Fiore Bavaro, Francesco Di Gennaro, Federica Salvati, Claudia Marotta, Annalisa Saracino, Rita Murri, and Massimo Fantoni. 2021. “Impact of SARS-CoV-2 Epidemic on Antimicrobial Resistance: A Literature Review.” *Viruses* 13 (11): 2110.
19	(Kanungo 2021)	Kanungo, Reba. 2021. “Antimicrobial Resistance and the COVID-19 Pandemic: A Double Threat.” *Indian Journal of Medical Microbiology* 39 (4): 401.
20	(Johnson 2021)	Johnson, Tess. 2021. “A Trade-off: Antimicrobial Resistance and COVID-19.” *Bioethics* 35 (9): 947–55.
21	(Despotovic et al. 2021)	Despotovic, Aleksa, Branko Milosevic, Andja Cirkovic, Ankica Vujovic, Ksenija Cucanic, Teodora Cucanic, and Goran Stevanovic. 2021. “The Impact of Covid-19 on the Profile of Hospital-Acquired Infections in Adult Intensive Care Units.” *Antibiotics* 10 (10): 1146.
22	(Lobie et al. 2021)	Lobie, Tekle Airgecho, Aklilu Abrham Roba, James Alexander Booth, Knut Ivan Kristiansen, Abraham Aseffa, Kirsten Skarstad, and Magnar Bjørås. 2021. “Antimicrobial Resistance: A Challenge Awaiting the Post-COVID-19 Era.” *International Journal of Infectious Diseases* 111 (October): 322–25. https://doi.org/10.1016/j.ijid.2021.09.003.
23	(Schouten et al. 2021)	Schouten, Jeroen, Jan De Waele, Christian Lanckohr, Despoina Koulenti, Nisrine Haddad, Nesrine Rizk, Fredrik Sjövall, and Souha S. Kanj. 2021. “Antimicrobial Stewardship in the ICU in COVID-19 Times: The Known Unknowns.” *International Journal of Antimicrobial Agents* 58 (4): 106409. https://doi.org/10.1016/j.ijantimicag.2021.106409.
24	(Ruiz-Garbajosa and Cantón 2021)	Ruiz-Garbajosa, P., and R. Cantón. 2021. “COVID-19: Impact on Prescribing and Antimicrobial Resistance.” *Revista Espanola de Quimioterapia : Publicacion Oficial de La Sociedad Espanola de Quimioterapia* 34 Suppl 1 (Suppl1): 63–68. https://doi.org/10.37201/req/s01.19.2021.
25	(Toro-Alzate, Hofstraat, and de Vries 2021)	Toro-Alzate, Luisa, Karlijn Hofstraat, and Daniel H. de Vries. 2021. “The Pandemic beyond the Pandemic: A Scoping Review on the Social Relationships between COVID-19 and Antimicrobial Resistance.” *International Journal of Environmental Research and Public Health* 18 (16). https://doi.org/10.3390/ijerph18168766.

26	(Andrews et al. 2021)	Andrews, Amelia, Emma L. Budd, Aoife Hendrick, Diane Ashiru-Oredope, Elizabeth Beech, Susan Hopkins, Sarah Gerver, Berit Muller-Pebody, and the AMU COVID-19 Stakeholder Group. 2021. "Surveillance of Antibacterial Usage during the COVID-19 Pandemic in England, 2020." *Antibiotics* 10 (7). https://doi.org/10.3390/antibiotics10070841.
27	(Haqqi et al. 2021)	Haqqi, Aleena, Usman Ayub Awan, Haroon Ahmed, and Muhammad Sohail Afzal. 2021. "Antimicrobial Resistance vs COVID-19: A Bigger Challenge in the Post-Pandemic Era!" *Journal of the Formosan Medical Association = Taiwan Yi Zhi* 120 (7): 1537–38. https://doi.org/10.1016/j.jfma.2021.03.003.
28	(Ansari et al. 2021)	Ansari, Shamshul, John P. Hays, Andrew Kemp, Raymond Okechukwu, Jayaseelan Murugaiyan, Mutshiene Deogratias Ekwanzala, Maria Josefina Ruiz Alvarez, Maneesh Paul-Satyaseela, Chidozie Declan Iwu, and Clara Balleste-Delpierre. 2021. "The Potential Impact of the COVID-19 Pandemic on Global Antimicrobial and Biocide Resistance: An AMR Insights Global Perspective." *JAC-Antimicrobial Resistance* 3 (2): dlab038.
29	(Kayarat, Khanna, and Sarkar 2021)	Kayarat, Bhavana, Puneet Khanna, and Soumya Sarkar. 2021. "Superadded Coinfections and Antibiotic Resistance in the Context of COVID-19: Where Do We Stand?" *Indian Journal of Critical Care Medicine : Peer-Reviewed, Official Publication of Indian Society of Critical Care Medicine* 25 (6): 699–703. https://doi.org/10.5005/jp-journals-10071-23855.
30	(Mazumder et al. 2021)	Mazumder, Payal, Ajay Kalamdhad, GG Tushara Chaminda, and Manish Kumar. 2021. "Coalescence of Co-Infection and Antimicrobial Resistance with SARS-CoV-2 Infection: The Blues of Post-COVID-19 World." *Case Studies in Chemical and Environmental Engineering* 3: 100093.
31	(Parveen, Yeasmin, and Molla 2020)	Parveen, Monira, Mahmuda Yeasmin, and Md. Maruf Ahmed Molla. 2020. "Antimicrobial Resistance, Evidences on Irrational Anti-Microbial Prescribing and Consumption during COVID-19 Pandemic and Possible Mitigation Strategies: A Bangladesh Perspective." *MedRxiv*. https://doi.org/10.1101/2020.10.09.20210377.
32	(Lucien et al. 2021)	Lucien, Mentor Ali Ber, Michael F. Canarie, Paul E. Kilgore, Gladzdin Jean-Denis, Natael Fénélon, Manise Pierre, Mauricio Cerpa, et al. 2021. "Antibiotics and Antimicrobial Resistance in the COVID-19 Era: Perspective from Resource-Limited Settings." *International Journal of Infectious Diseases* 104 (March): 250–54. https://doi.org/10.1016/j.ijid.2020.12.087.
33	(Knight et al. 2021)	Knight, Gwenan M, Rebecca E Glover, C Finn McQuaid, Ioana D Olaru, Karin Gallandat, Quentin J Leclerc, Naomi M Fuller, et al. 2021.

		"Antimicrobial Resistance and COVID-19: Intersections and Implications." Edited by Vaughn S Cooper and George H Perry. *ELife* 10 (February): e64139. https://doi.org/10.7554/eLife.64139.
34	(Ukuhor 2020)	Ukuhor, Hyacinth O. 2020. "The Interrelationships between Antimicrobial Resistance, COVID-19, Past, and Future Pandemics." *Journal of Infection and Public Health*.
35	(Rodríguez-Álvarez et al. 2021)	Rodríguez-Álvarez, Mauricio, Yolanda López-Vidal, José Luis Soto-Hernández, María Guadalupe Miranda-Novales, Karen Flores-Moreno, and Samuel Ponce de León-Rosales. 2021. "COVID-19: Clouds Over the Antimicrobial Resistance Landscape." *Archives of Medical Research* 52 (1): 123–26. https://doi.org/10.1016/j.arcmed.2020.10.010.
36	(Yam 2020)	Yam, Esabelle Lo Yan. 2020. "COVID-19 Will Further Exacerbate Global Antimicrobial Resistance." *Journal of Travel Medicine* 27 (6): taaa098. https://doi.org/10.1093/jtm/taaa098.
37	(Clancy and Nguyen 2020)	Clancy, Cornelius J, and M Hong Nguyen. 2020. "Coronavirus Disease 2019, Superinfections, and Antimicrobial Development: What Can We Expect?" *Clinical Infectious Diseases* 71 (10): 2736–43. https://doi.org/10.1093/cid/ciaa524.
38	(Cantón, Gijón, and Ruiz-Garbajosa 2020)	Cantón, Rafael, Desirèe Gijón, and Patricia Ruiz-Garbajosa. 2020. "Antimicrobial Resistance in ICUs: An Update in the Light of the COVID-19 Pandemic." *Current Opinion in Critical Care* 26 (5). https://journals.lww.com/co-
39	(Clancy, Buehrle, and Nguyen 2020)	Clancy, Cornelius J., Deanna J. Buehrle, and M. Hong Nguyen. 2020. "PRO: The COVID-19 Pandemic Will Result in Increased Antimicrobial Resistance Rates." *JAC-Antimicrobial Resistance* 2 (3): dlaa049.
40	(Collignon and Beggs 2020)	Collignon, Peter, and John J Beggs. 2020. "CON: COVID-19 Will Not Result in Increased Antimicrobial Resistance Prevalence." *JAC-Antimicrobial Resistance* 2 (3): dlaa051. https://doi.org/10.1093/jacamr/dlaa051
41	(Getahun et al. 2020b)	Getahun, Haileyesus, Ingrid Smith, Kavita Trivedi, Sarah Paulin, and Hanan H. Balkhy. 2020a. "Tackling Antimicrobial Resistance in the COVID-19 Pandemic." *Bulletin of the World Health Organization* 98 (7): 442.
42	(Rawson, Moore, Castro-Sanchez, et al. 2020)	Rawson, Timothy M, Luke S P Moore, Enrique Castro-Sanchez, Esmita Charani, Frances Davies, Giovanni Satta, Matthew J Ellington, and Alison H Holmes. 2020. "COVID-19 and the Potential Long-Term Impact on Antimicrobial Resistance." *Journal of Antimicrobial Chemotherapy* 75 (7): 1681–84. https://doi.org/10.1093/jac/dkaa194.

43	(Murray 2020)	Murray, Aimee K. 2020. "The Novel Coronavirus COVID-19 Outbreak: Global Implications for Antimicrobial Resistance." *Frontiers in Microbiology* 11: 1020.
44	(Majumder et al. 2020)	Majumder, Md Anwarul Azim, Sayeeda Rahman, Damian Cohall, Ambadasu Bharatha, Keerti Singh, Mainul Haque, and Marquita Gittens-St Hilaire. 2020. "Antimicrobial Stewardship: Fighting Antimicrobial Resistance and Protecting Global Public Health." *Infection and Drug Resistance* 13: 4713–38. https://doi.org/10.2147/IDR.S290835.
45	(Iwu et al. 2020)	Iwu, Chinwe Juliana, Portia Jordan, Ishmael Festus Jaja, Chidozie Declan Iwu, and Charles Shey Wiysonge. 2020. "Treatment of COVID-19: Implications for Antimicrobial Resistance in Africa." *The Pan African Medical Journal* 35 (Suppl 2): 119. https://doi.org/10.11604/pamj.supp.2020.35.23713.
46	(Pulia et al. 2020)	Pulia, Michael S., Ian Wolf, Lucas T. Schulz, Aurora Pop-Vicas, Rebecca J. Schwei, and Peter K. Lindenauer. 2020. "COVID-19: An Emerging Threat to Antibiotic Stewardship in the Emergency Department." *The Western Journal of Emergency Medicine* 21 (5): 1283–86. https://doi.org/10.5811/westjem.2020.7.48848.
47	(Baggs et al. 2020)	Baggs, James, Ashley N. Rose, Natalie L. McCarthy, Hannah Wolford, Arjun Srinivasan, John A. Jernigan, and Sujan C. Reddy. 2022. "Antibiotic-Resistant Infections Among Inpatients with Coronavirus Disease 2019 (COVID-19) in US Hospitals." *Clinical Infectious Diseases* 75 (Supplement_2): S294–97.
48	(Yusuf and Sarkinfada 2021)	Yusuf, Ibrahim, and Faruk Sarkinfada. 2021. "Gaps in the Implementation of COVID-19 Mitigation Measures Could Lead to Development of New Strains of Antimicrobial Resistant Pathogens: Nigerian Perspective." *The Pan African Medical Journal* 40: 12. https://doi.org/10.11604/pamj.2021.40.12.23274.
49	(Nieuwlaat et al. 2021)	Nieuwlaat, Robby, Lawrence Mbuagbaw, Dominik Mertz, Lori L. Burrows, Dawn ME Bowdish, Lorenzo Moja, Gerard D. Wright, and Holger J. Schünemann. 2021. "Coronavirus Disease 2019 and Antimicrobial Resistance: Parallel and Interacting Health Emergencies." *Clinical Infectious Diseases* 72 (9): 1657–59.
50	(Sulayyim et al. 2022)	Sulayyim, Hadi Jaber Al, Rohani Ismail, Abdullah Al Hamid, and Noraini Abdul Ghafar. 2022. "Antibiotic Resistance during COVID-19: A Systematic Review." *International Journal of Environmental Research and Public Health* 19 (19). https://doi.org/10.3390/ijerph191911931.

51	(Muflih et al. 2021)	Muflih, Suhaib M., Sayer Al-Azzam, Reema A. Karasneh, Barbara R. Conway, and Mamoon A. Aldeyab. 2021. “Public Health Literacy, Knowledge, and Awareness Regarding Antibiotic Use and Antimicrobial Resistance during the COVID-19 Pandemic: A Cross-Sectional Study.” *Antibiotics* 10 (9). https://doi.org/10.3390/antibiotics10091107.
52	(Adebisi, Jimoh, et al. 2021)	Adebisi, Yusuff Adebayo, Nafisat Dasola Jimoh, Isaac Olushola Ogunkola, Theogene Uwizeyimana, Alaka Hassan Olayemi, Nelson Ashinedu Ukor, and Don Eliseo Lucero-Prisno. 2021. “The Use of Antibiotics in COVID-19 Management: A Rapid Review of National Treatment Guidelines in 10 African Countries.” *Tropical Medicine and Health* 49 (1): 1–5.
53	(Akhtar et al. 2021)	Akhtar, Hashaam, Samar Akhtar, Fazal-Ul Rahman, Maham Afridi, Sundas Khalid, Sabahat Ali, Nasim Akhtar, Yousef S Khader, Hamaad Ahmad, and Muhammad Mujeeb Khan. 2021. “An Overview of the Treatment Options Used for the Management of COVID-19 in Pakistan: Retrospective Observational Study.” *JMIR Public Health Surveill* 7 (5): e28594. https://doi.org/10.2196/28594.
54	(Hayat et al. 2021)	Hayat, Khezar, Zia Ul Mustafa, Muhammad Nabeel Ikram, Muhammad Ijaz-Ul-Haq, Irum Noor, Muhammad Fawad Rasool, Hafiz Muhammad Ishaq, Anees Ur Rehman, Syed Shahzad Hasan, and Yu Fang. 2021. “Perception, Attitude, and Confidence of Physicians About Antimicrobial Resistance and Antimicrobial Prescribing Among COVID-19 Patients: A Cross-Sectional Study From Punjab, Pakistan.” *Frontiers in Pharmacology* 12: 794453. https://doi.org/10.3389/fphar.2021.794453.
55	(Mahmoudi 2020, 19)	Mahmoudi, Hassan. 2020. “Bacterial Co-Infections and Antibiotic Resistance in Patients with COVID-19.” *GMS Hygiene and Infection Control* 15: Doc35. https://doi.org/10.3205/dgkh000370.
56	(Rusic et al. 2021)	Rusic, Doris, Marino Vilovic, Josipa Bukic, Dario Leskur, Ana Seselja Perisin, Marko Kumric, Dinko Martinovic, Ana Petric, Darko Modun, and Josko Bozic. 2021. “Implications of COVID-19 Pandemic on the Emergence of Antimicrobial Resistance: Adjusting the Response to Future Outbreaks.” *Life* 11 (3). https://doi.org/10.3390/life11030220.
57	(Ashiru-Oredope et al. 2021)	Ashiru-Oredope, Diane, Frances Kerr, Stephen Hughes, Jonathan Urch, Marisa Lanzman, Ting Yau, Alison Cockburn, et al. 2021. “Assessing the Impact of COVID-19 on Antimicrobial Stewardship Activities/Programs in the United Kingdom.” *Antibiotics* 10 (2). https://doi.org/10.3390/antibiotics10020110.

58	(Rezasoltani et al. 2020)	Rezasoltani, Sama, Abbas Yadegar, Behzad Hatami, Hamid Asadzadeh Aghdaei, and Mohammad Reza Zali. 2020. "Antimicrobial Resistance as a Hidden Menace Lurking Behind the COVID-19 Outbreak: The Global Impacts of Too Much Hygiene on AMR." *Frontiers in Microbiology* 11. https://www.frontiersin.org/articles/10.3389/fmicb.2020.590683.
59	(Zhang et al. 2021)	Zhang, Airong, Elizabeth V. Hobman, Paul De Barro, Asaesja Young, David J. Carter, and Mitchell Byrne. 2021. "Self-Medication with Antibiotics for Protection against COVID-19: The Role of Psychological Distress, Knowledge of, and Experiences with Antibiotics." *Antibiotics* 10 (3): 232.
60	(Lai et al. 2021)	Lai, Chih-Cheng, Shey-Ying Chen, Wen-Chien Ko, and Po-Ren Hsueh. 2021. "Increased Antimicrobial Resistance during the COVID-19 Pandemic." *International Journal of Antimicrobial Agents* 57 (4): 106324. https://doi.org/10.1016/j.ijantimicag.2021.106324.
61	(Rawson, Moore, Zhu, et al. 2020)	Rawson, Timothy M, Luke S P Moore, Nina Zhu, Nishanthy Ranganathan, Keira Skolimowska, Mark Gilchrist, Giovanni Satta, Graham Cooke, and Alison Holmes. 2020. "Bacterial and Fungal Coinfection in Individuals With Coronavirus: A Rapid Review To Support COVID-19 Antimicrobial Prescribing." *Clinical Infectious Diseases* 71 (9): 2459–68. https://doi.org/10.1093/cid/ciaa530.
62	(Langford et al. 2021)	Langford, Bradley J., Miranda So, Sumit Raybardhan, Valerie Leung, Jean-Paul R. Soucy, Duncan Westwood, Nick Daneman, and Derek R. MacFadden. 2021. "Antibiotic Prescribing in Patients with COVID-19: Rapid Review and Meta-Analysis." *Clinical Microbiology and Infection* 27 (4): 520–31. https://doi.org/10.1016/j.cmi.2020.12.018.
63	(Ghosh, Bornman, and Zafer 2021)	Ghosh, Soumya, Charné Bornman, and Mai M. Zafer. 2021. "Antimicrobial Resistance Threats in the Emerging COVID-19 Pandemic: Where Do We Stand?" *Journal of Infection and Public Health* 14 (5): 555–60. https://doi.org/10.1016/j.jiph.2021.02.011.
64	(Stefanini et al. 2021, 2)	Stefanini, Irene, Giuseppe De Renzi, Elisa Foddai, Elisa Cordani, and Barbara Mognetti. 2021. "Profile of Bacterial Infections in COVID-19 Patients: Antimicrobial Resistance in the Time of SARS-CoV-2." *Biology* 10 (9). https://doi.org/10.3390/biology10090822.
65	(Devi 2020)	Devi, Sharmila. 2020. "No Time to Lower the Guard on AMR." *The Lancet Microbe* 1 (5): e198. https://doi.org/10.1016/S2666-5247(20)30129-4.

66	(O’Toole 2021)	O’Toole, Ronan F. 2021. “The Interface between COVID-19 and Bacterial Healthcare-Associated Infections.” *Clinical Microbiology and Infection : The Official Publication of the European Society of Clinical Microbiology and Infectious Diseases* 27 (12): 1772–76. https://doi.org/10.1016/j.cmi.2021.06.001.
67	(Adebisi, Alaran, et al. 2021)	Adebisi, Yusuff Adebayo, Aishat Jumoke Alaran, Melody Okereke, Gabriel Ilerioluwa Oke, Oladunni Abimbola Amos, Omotayo Carolyn Olaoye, Iyiola Oladunjoye, Azeez Yusuff Olanrewaju, Nelson Ashinedu Ukor, and Don Eliseo 3rd Lucero-Prisno. 2021. “COVID-19 and Antimicrobial Resistance: A Review.” *Infectious Diseases* 14: 11786337211033870.
68	(Fattorini et al. 2020)	Fattorini, Lanfranco, Roberta Creti, Carla Palma, and Annalisa Pantosti. 2020. “Bacterial Coinfections in COVID-19: An Underestimated Adversary.” *Annali Dell’Istituto Superiore Di Sanita* 56 (3): 359–64. https://doi.org/10.4415/ANN_20_03_14.
69	(Livermore 2021, 19)	Livermore, David M. 2021. “Antibiotic Resistance during and beyond COVID-19.” *JAC-Antimicrobial Resistance* 3 (Suppl 1): i5–16. https://doi.org/10.1093/jacamr/dlab052.
70	(Martin et al. 2021)	Martin, Ellen, Marie Philbin, Gerry Hughes, Colm Bergin, and Alida Fe Talento. 2021. “Antimicrobial Stewardship Challenges and Innovative Initiatives in the Acute Hospital Setting during the COVID-19 Pandemic.” *Journal of Antimicrobial Chemotherapy* 76 (1): 272–75. https://doi.org/10.1093/jac/dkaa400.
71	(Baskaran et al. 2021)	Baskaran, Vadsala, Hannah Lawrence, Louise E. Lansbury, Karmel Webb, Shahideh Safavi, Nurul I. Zainuddin, Tausif Huq, et al. 2021. “Co-Infection in Critically Ill Patients with COVID-19: An Observational Cohort Study from England.” *Journal of Medical Microbiology* 70 (4). https://doi.org/10.1099/jmm.0.001350.
72	(Dr. Apoorva Mathur 2021)	Dr. Apoorva Mathur, Prof. Y.K. Gupta. 2021. “Antimicrobials Overuse in COVID-19: A Silent Pandemic of Antimicrobial Resistance.” *The Indian Practitioner* 74 (5).
73	(Balasegaram 2021)	Balasegaram, Manica. 2021. “Learning from COVID-19 to Tackle Antibiotic Resistance.” *ACS Infectious Diseases* 7 (4): 693–94. https://doi.org/10.1021/acsinfecdis.1c00079.
74	(Founou et al. 2021)	Founou, Raspail C., Ariel J. Blocker, Michel Noubom, Cedrice Tsayem, Siméon P. Choukem, Maarten Van Dongen, and Luria L. Founou. 2021. “The COVID-19 Pandemic: A Threat to Antimicrobial Resistance Containment.” *Future Science OA* 7 (8): FSO736. https://doi.org/10.2144/fsoa-2021-0012.
75	(Ami Patel 2021, 19)	Patel, Ami. 2021. “Tackling Antimicrobial Resistance in the Shadow of COVID-19.” *MBio* 12 (4): e0047321. https://doi.org/10.1128/mBio.00473-21.

76	(Mahalmani et al. 2021)	Mahalmani, Vidya, J. Kumaravel, Manav Jain, Ajay Prakash, and Bikash Medhi. 2021. “Antimicrobial Resistance: An Unseen Threat Prowling behind the COVID-19 Outbreak.” *Indian Journal of Pharmacology* 53 (3): 187–91. https://doi.org/10.4103/ijp.ijp_430_21.
77	(Mayi et al. 2021, 19)	Mayi, Bindu S., Manda Mainville, Rida Altaf, Michelle Lanspa, Sheel Vaniawala, Thomas A. Ollerhead, and Aarti Raja. 2021. “A Crucial Role for Antimicrobial Stewardship in the Midst of COVID-19.” *Journal of Microbiology & Biology Education* 22 (1). https://doi.org/10.1128/jmbe.v22i1.2285.
78	(Subramanya et al. 2021)	Subramanya, Supram Hosuru, Daniel M. Czyż, Krishna Prasad Acharya, and Hilary Humphreys. 2021. “The Potential Impact of the COVID-19 Pandemic on Antimicrobial Resistance and Antibiotic Stewardship.” *Virusdisease* 32 (2): 330–37. https://doi.org/10.1007/s13337-021-00695-2.
79	(Lynch, Mahida, and Gray 2020)	Lynch, C., N. Mahida, and J. Gray. 2020. “Antimicrobial Stewardship: A COVID Casualty?” *The Journal of Hospital Infection* 106 (3): 401–3. https://doi.org/10.1016/j.jhin.2020.10.002
80	(Monnet and Harbarth 2020)	Monnet, Dominique L., and Stephan Harbarth. 2020. “Will Coronavirus Disease (COVID-19) Have an Impact on Antimicrobial Resistance?” *Euro Surveillance : Bulletin Europeen Sur Les Maladies Transmissibles = European Communicable Disease Bulletin* 25 (45). https://doi.org/10.2807/1560-7917.ES.2020.25.45.2001886.
81	(Ruiz 2021)	Ruiz, J. 2021. “Enhanced Antibiotic Resistance as a Collateral COVID-19 Pandemic Effect?” *The Journal of Hospital Infection* 107 (January): 114–15. https://doi.org/10.1016/j.jhin.2020.11.010.
82	(Rizk et al. 2021)	Rizk, Nesrine A., Rima Moghnieh, Nisrine Haddad, Marie-Claire Rebeiz, Rony M. Zeenny, Joya-Rita Hindy, Gabriella Orlando, and Souha S. Kanj. 2021. “Challenges to Antimicrobial Stewardship in the Countries of the Arab League: Concerns of Worsening Resistance during the COVID-19 Pandemic and Proposed Solutions.” *Antibiotics* 10 (11): 1320.
83	(Al-Azzam et al. 2021)	Al-Azzam, Sayer, Nizar Mahmoud Mhaidat, Hayaa A. Banat, Mohammad Alfaour, Dana Samih Ahmad, Arno Muller, Adi Al-Nuseirat, Elizabeth A. Lattyak, Barbara R. Conway, and Mamoon A. Aldeyab. 2021. “An Assessment of the Impact of Coronavirus Disease (COVID-19) Pandemic on National Antimicrobial Consumption in Jordan.” *Antibiotics (Basel, Switzerland)* 10 (6). https://doi.org/10.3390/antibiotics10060690.

84	(Chibabhai et al. 2020)	Chibabhai, V., A. G. Duse, O. Perovic, and G. A. Richards. 2020. "Collateral Damage of the COVID-19 Pandemic: Exacerbation of Antimicrobial Resistance and Disruptions to Antimicrobial Stewardship Programmes?" *South African Medical Journal = Suid-Afrikaanse Tydskrif Vir Geneeskunde* 110 (7): 572–73. https://doi.org/10.7196/SAMJ.2020.v110i7.14917.
85	(Tan et al. 2021)	Tan, Sock Hoon, Tat Ming Ng, Hui Lin Tay, Min Yi Yap, Shi Thong Heng, Audrey Yong Xin Loo, Christine B. Teng, and Tau Hong Lee. 2021. "A Point Prevalence Survey to Assess Antibiotic Prescribing in Patients Hospitalized with Confirmed and Suspected Coronavirus Disease 2019 (COVID-19)."
86	(Spernovasilis et al. 2021)	Spernovasilis, Nikolaos, Despo Ierodiakonou, Christos Spanias, Anna Mathioudaki, Petros Ioannou, Emmanouil C. Petrakis, and Diamantis P. Kofteridis. 2021. "Doctors' Perceptions, Attitudes and Practices towards the Management of Multidrug-Resistant Organism Infections after the Implementation of an Antimicrobial Stewardship Programme during the COVID-19 Pandemic." *Tropical Medicine and Infectious Disease* 6 (1). https://doi.org/10.3390/tropicalmed6010020.
87	(Ul Mustafa et al. 2021)	Ul Mustafa, Zia, Muhammad Salman, Mamoon Aldeyab, Chia Siang Kow, and Syed Shahzad Hasan. 2021. "Antimicrobial Consumption among Hospitalized Patients with COVID-19 in Pakistan." *SN Comprehensive Clinical Medicine* 3 (8): 1691–95. https://doi.org/10.1007/s42399-021-00966-5.
88	(Liu et al. 2021)	Liu, Hans H., David Yaron, Amanda Stahl Piraino, and Luciano Kapelusznik. 2021. "Bacterial and Fungal Growth in Sputum Cultures from 165 COVID-19 Pneumonia Patients Requiring Intubation: Evidence for Antimicrobial Resistance Development and Analysis of Risk Factors." *Annals of Clinical Microbiology and Antimicrobials* 20 (1): 69. https://doi.org/10.1186/s12941-021-00472-5.
89	(Khurana et al. 2021)	Khurana, Surbhi, Parul Singh, Neha Sharad, Vandana V. Kiro, Neha Rastogi, Amit Lathwal, Rajesh Malhotra, Anjan Trikha, and Purva Mathur. 2021. "Profile of Co-Infections & Secondary Infections in COVID-19 Patients at a Dedicated COVID-19 Facility of a Tertiary Care Indian Hospital: Implication on Antimicrobial Resistance." *Indian Journal of Medical Microbiology* 39 (2): 147–53. https://doi.org/10.1016/j.ijmmb.2020.10.014.
90	(Al-Hadidi et al. 2021)	Al-Hadidi, Sara H., Hashim Alhussain, Hamad Abdel Hadi, Alreem Johar, Hadi M. Yassine, Asmaa A. Al Thani, and Nahla O. Eltai. 2021. "The Spectrum of Antibiotic Prescribing during COVID-19 Pandemic: A Systematic Literature Review." *Microbial Drug Resistance* 27 (12): 1705–25.

91	(Jahanshahlou and Hosseini 2021)	Jahanshahlou, Farid, and Mohammad-Salar Hosseini. 2021. "Antibiotic Resistance: A Disregarded Concern for Misuse of Azithromycin in COVID-19 Treatment." *Journal of Research in Medical Sciences : The Official Journal of Isfahan University of Medical Sciences* 26: 101. https://doi.org/10.4103/jrms.JRMS_1124_20.
92	(Grau et al. 2021)	Grau, Santiago, Daniel Echeverria-Esnal, Silvia Gómez-Zorrilla, Maria Eugenia Navarrete-Rouco, Joan Ramon Masclans, Merce Espona, Maria Pilar Gracia-Arnillas, et al. 2021. "Evolution of Antimicrobial Consumption During the First Wave of COVID-19 Pandemic." *Antibiotics (Basel, Switzerland)* 10 (2). https://doi.org/10.3390/antibiotics10020132.
93	(Laupland, Collignon, and Schwartz 2021)	Laupland, K.B., P.J. Collignon, and I.S. Schwartz. 2021. "Sleeping with the Enemy: Will the Covid-19 Pandemic Turn the Tide of Antimicrobial-Resistant Infections?" *JAMMI* 6 (3): 177–80. https://doi.org/10.3138/JAMMI-2021-05-28.
94	(Dahal et al. 2021)	Dahal, Deepa, Swarna Sundar, Ravina Kullar, Neda Milevska-Kostova, and Karen Dindial. 2021. "Antimicrobial Resistance during the COVID-19 Pandemic: The Missing Patient Perspective." *JAC-Antimicrobial Resistance* 3 (1): dlab030. https://doi.org/10.1093/jacamr/dlab030.
95	(Owoichow et al. 2021)	Owoichow, O, K Tapela, ALD Zune, NN Nghochuzie, A Isawumi, and L Mosi. 2021. "Suboptimal Antimicrobial Stewardship in the COVID-19 Era: Is Humanity Staring at a Postantibiotic Future?" *FUTURE MICROBIOLOGY* 16 (12): 919–25. https://doi.org/10.2217/fmb-2021-0008.
96	(Cama et al. 2021)	Cama, J., R. Leszczynski, P. K. Tang, A. Khalid, V. Lok, C. G. Dowson, and A. Ebata. 2021. "To Push or To Pull? In a Post-COVID World, Supporting and Incentivizing Antimicrobial Drug Development Must Become a Governmental Priority." *ACS Infectious Diseases* 7 (8): 2029–42. https://doi.org/10.1021/acsinfecdis.0c00681.
97	(Saini et al. 2021)	Saini, Vikas, Charu Jain, Narendra Pal Singh, Ahmad Alsulimani, Chhavi Gupta, Sajad Ahmad Dar, Shafiul Haque, and Shukla Das. 2021. "Paradigm Shift in Antimicrobial Resistance Pattern of Bacterial Isolates during the COVID-19 Pandemic." *Antibiotics* 10 (8). https://doi.org/10.3390/antibiotics10080954.
98	(Alvi et al. 2021)	Alvi, Mohammad Zaheer-Ud-Din, Mudassar Sattar, Fatima Amir Cheema, Usman Ayub Awan, Muhammad Nauman Aftab, Zaheer Hussain Shah, and Muhammad Sohail Afzal. 2021. "COVID-19 and Emergence of Antimicrobial Resistance: A Most Neglected Aspect of Health Emergency in Pakistan." *Diabetes & Metabolic Syndrome* 15 (4): 102179. https://doi.org/10.1016/j.dsx.2021.06.016.

99	(Mah-E-Muneer et al. 2021)	Mah-E-Muneer, Syeda, Md Zakiul Hassan, Md Abdullah Al Jubayer Biswas, Fahmida Rahman, Zubair Akhtar, Pritimoy Das, Md Ariful Islam, and Fahmida Chowdhury. 2021. “Use of Antimicrobials among Suspected COVID-19 Patients at Selected Hospitals, Bangladesh: Findings from the First Wave of COVID-19 Pandemic.” *Antibiotics (Basel, Switzerland)* 10 (6). https://doi.org/10.3390/antibiotics10060738.
100	(Dutta and Haque 2021)	Dutta, Siddhartha, and Mainul Haque. 2021. “Covid-19: Questions of Antimicrobial Resistance.” *Bangladesh Journal of Medical Science*. https://doi.org/10.3329/bjms.v20i2.51527.
101	(Karnoukh and Lazareva 2021)	Karnoukh, Konstantin I., and Natalia B. Lazareva. 2021. “Analysis of the Antibiotic Consumption on the Backdrop of the COVID-19 Pandemic: Hospital Level.” *Meditsinskiy Sovet*. https://doi.org/10.21518/2079-701X-2021-16-118-128.
102	(Gaspar et al. 2021)	Gaspar, Gilberto Gambero, Lécio Rodrigues Ferreira, Cinara Silva Feliciano, Cláudio Penido Campos Júnior, Fabiana Murad Rossin Molina, Andrea Cristina Soares Vendruscolo, Giovana Marcão Araújo Bradan, Nátali Artal Padovani Lopes, Roberto Martinez, and Valdes Roberto Bollela. 2021. “Pre-and Post-Covid-19 Evaluation of Antimicrobial Susceptibility for Healthcare-Associated Infections in the Intensive Care Unit of a Tertiary Hospital.” *Revista Da Sociedade Brasileira de Medicina Tropical*. https://doi.org/10.1590/0037-8682-0090-2021
103	(Seabra et al. 2021)	Seabra, Gabriela, Roberta Ferreira Ventura Mendes, Luiz Felipe Vieira Dos Santos Amorim, Ingrid Vianez Peregrino, Marta Helena Branquinha, André Luis Souza Dos Santos, and Ana Paula Ferreira Nunes. 2021. “Azithromycin Use in Covid-19 Patients: Implications on the Antimicrobial Resistance.” *Current Topics in Medicinal Chemistry*. https://doi.org/10.2174/156802662108210319145317.
104	(Álvarez-Moreno, Valderrama-Beltrán, and Rodriguez-Morales 2021)	Álvarez-Moreno, Carlos, Sandra Valderrama-Beltrán, and Alfonso J. Rodriguez-Morales. 2021. “Implications of Antibiotic Use during the Covid-19 Pandemic: The Example of Associated Antimicrobial Resistance in Latin America.” *Antibiotics*. https://doi.org/10.3390/antibiotics10030328.
105	(Rahube 2021)	Rahube, Teddie O. 2021. “Cease Approach for Combating Covid-19, Antimicrobial Resistance, and Future Microbial Threats.” *Canadian Journal of Microbiology*. https://doi.org/10.1139/cjm-2020-0452.
106	(Razzaque 2021)	Razzaque, Mohammed S. 2021. “Exacerbation of Antimicrobial Resistance: Another Casualty of the COVID-19 Pandemic?” *Expert Review of Anti-Infective Therapy*. https://doi.org/10.1080/14787210.2021.1865802.

107	(Miranda et al. 2020)	Miranda, Carla, Vanessa Silva, Rosa Capita, Carlos Alonso-Calleja, Gilberto Igrejas, and Patrícia Poeta. 2020. "Implications of Antibiotics Use during the COVID-19 Pandemic: Present and Future." *Journal of Antimicrobial Chemotherapy*. https://doi.org/10.1093/jac/dkaa350.
108	(Arshad et al. 2020)	Arshad, M., S.F. Mahmood, M. Khan, and R. Hasan. 2020. "Covid -19, Misinformation, and Antimicrobial Resistance." *The BMJ* 371. https://doi.org/10.1136/bmj.m4501.
109	(Guisado-Gil et al. 2020)	Guisado-Gil, Ana Belen, Carmen Infante-Domínguez, Germán Peñalva, Julia Praena, Cristina Roca, María Dolores Navarro-Amuedo, Manuela Aguilar-Guisado, et al. 2020. "Impact of the COVID-19 Pandemic on Antimicrobial Consumption and Hospital-Acquired Candidemia and Multidrug-Resistant Bloodstream Infections." *Antibiotics*. https://doi.org/10.3390/antibiotics9110816.
110	(Wilson et al. 2020)	Wilson, Lindsay A., Susan Rogers Van Katwyk, Patrick Fafard, A.M. Viens, and Steven J. Hoffman. 2020. "Lessons Learned from COVID-19 for the Post-Antibiotic Future." *Globalization and Health*. https://doi.org/10.1186/s12992-020-00623-x.
111	(CantoÂ N, GijoÂn, and Ruiz-Garbajosa 2020)	CantoÂ N, Rafael, Desireè GijoÂn, and Patricia Ruiz-Garbajosa. 2020. "Antimicrobial Resistance in ICUs: An Update in the Light of the COVID-19 Pandemic." *Current Opinion in Critical Care*. https://doi.org/10.1097/MCC.0000000000000755.
112	(Li et al. 2020)	Li, Jie, Junwei Wang, Yi Yang, Peishan Cai, Jingchao Cao, Xuefeng Cai, and Yu Zhang. 2020. "Etiology and Antimicrobial Resistance of Secondary Bacterial Infections in Patients Hospitalized with COVID-19 in Wuhan, China: A Retrospective Analysis." *Antimicrobial Resistance and Infection Control*. https://doi.org/10.1186/s13756-020-00819-1.
113	(Van Duin, Barlow, and Nathwani 2020)	Van Duin, David, Gavin Barlow, and Dilip Nathwani. 2020. "The Impact of the COVID-19 Pandemic on Antimicrobial Resistance: A Debate." *JAC-Antimicrobial Resistance*. https://doi.org/10.1093/jacamr/dlaa053.
114	(Rossato, Negrão, and Simionatto 2020)	Rossato, Luana, Fábio Juliano Negrão, and Simone Simionatto. 2020. "Could the COVID-19 Pandemic Aggravate Antimicrobial Resistance?" *American Journal of Infection Control*. https://doi.org/10.1016/j.ajic.2020.06.192.
115	(Hsu 2020)	Hsu, Jeremy. 2020. "How Covid-19 Is Accelerating the Threat of Antimicrobial Resistance." *BMJ* 369.<br>Jampani, Mahesh, and Sujith J. Chandy. 2021. "Increased Antimicrobial Use during COVID

116	(Hashmi et al. 2020)	Hashmi, Furqan Khurshid, Naveel Atif, Usman Rashid Malik, Fahad Saleem, Zineb Riboua, Mohamed Azmi Hassali, Muhammad Hammad Butt, Tauqeer Hussain Mallhi, and Yusra Habib Khan. 2020. "In Pursuit of COVID-19 Treatment Strategies: Are We Triggering Antimicrobial Resistance?" *Disaster Medicine and Public Health Preparedness*. https://doi.org/10.1017/dmp.2020.492.
117	(Egyir, Obeng-Nkrumah, and Kyei 2020)	Egyir, Beverly, Noah Obeng-Nkrumah, and George B. Kyei. 2020. "COVID-19 Pandemic and Antimicrobial Resistance: Another Call to Strengthen Laboratory Diagnostic Capacity in Africa." *African Journal of Laboratory Medicine*. https://doi.org/10.4102/ajlm.v9i1.1302.
118	(Ramadan et al. 2020)	Ramadan, Haidi Karam-Allah, Manal A. Mahmoud, Mohamed Zakaria, Aburahma, Amal A. Elkhawaga, Mohamed A. El-Mokhtar, Ibrahim M. Sayed, Amal Hosni, Sahar M. Hassany, and Mohammed A. Medhat. 2020. "Predictors of Severity and Co-Infection Resistance Profile in COVID-19 Patients: First Report from Upper Egypt." *Infection and Drug Resistance*. https://doi.org/10.2147/IDR.S272605.
119	(Vaillancourt and Jorth 2020, 19)	Vaillancourt, Mylene, and Peter Jorth. 2020. "The Unrecognized Threat of Secondary Bacterial Infections with COVID-19." *MBIO* 11 (4). https://doi.org/10.1128/mBio.01806-20.
120	(Assi et al. 2021)	Assi, Mariam, Salma Abbas, Priya Nori, Michelle Doll, Emily Godbout, Gonzalo Bearman, and Michael P. Stevens. 2021. "Infection Prevention and Antimicrobial Stewardship Program Collaboration During the COVID-19 Pandemic: A Window of Opportunity." *Current Infectious Disease Reports* 23 (10): 15. https://doi.org/10.1007/s11908-021-00759-w.
121	(Kost 2021)	Kost, Gerald J. 2021. "Geospatial Spread of Antimicrobial Resistance, Bacterial and Fungal Threats to Coronavirus Infectious Disease 2019 (COVID-19) Survival, and Point-of-Care Solutions." *Archives of Pathology & Laboratory Medicine* 145 (2): 145–67. https://doi.org/10.5858/arpa.2020-0284-RA.
122	(Ashka Patel et al. 2021)	Patel, Ashka, Michele Emerick, Marie K. Cabunoc, Michelle H. Williams, Michael Anne Preas, Gregory Schrank, Ronald Rabinowitz, Paul Luethy, J. Kristie Johnson, and Surbhi Leekha. 2021. "Rapid Spread and Control of Multidrug-Resistant Gram-Negative Bacteria in COVID-19 Patient Care Units." *Emerging Infectious Diseases* 27 (4): 1234–37. https://doi.org/10.3201/eid2704.204036.
123	(B. Chen et al. 2021)	Chen, Bo, Jie Han, Han Dai, and Puqi Jia. 2021. "Biocide-Tolerance and Antibiotic-Resistance in Community Environments and Risk of Direct Transfers to Humans: Unintended Consequences of

		Community-Wide Surface Disinfecting during COVID-19?" *Environmental Pollution (Barking, Essex : 1987)* 283 (August): 117074. https://doi.org/10.1016/j.envpol.2021.117074.
124	(Zhou et al. 2021)	Zhou, Jie, Jonathan A. Otter, James R. Price, Cristina Cimpeanu, Danel Meno Garcia, James Kinross, Piers R. Boshier, et al. 2021. "Investigating Severe Acute Respiratory Syndrome Coronavirus 2 (SARS-CoV-2) Surface and Air Contamination in an Acute Healthcare Setting During the Peak of the Coronavirus Disease 2019 (COVID-19) Pandemic in London." *Clinical Infectious Diseases : An Official Publication of the Infectious Diseases Society of America* 73 (7): e1870–77. https://doi.org/10.1093/cid/ciaa905.
125	(Chedid et al. 2021)	Chedid, Marie, Rami Waked, Elie Haddad, Nabil Chetata, Gebrael Saliba, and Jacques Choucair. 2021. "Antibiotics in Treatment of COVID-19 Complications: A Review of Frequency, Indications, and Efficacy." *Journal of Infection and Public Health* 14 (5): 570–76. https://doi.org/10.1016/j.jiph.2021.02.001.
126	(Vijay et al. 2021)	Vijay, Sonam, Nitin Bansal, Brijendra Kumar Rao, Balaji Veeraraghavan, Camilla Rodrigues, Chand Wattal, Jagdish Prasad Goyal, et al. 2021. "Secondary Infections in Hospitalized COVID-19 Patients: Indian Experience." *Infection and Drug Resistance* 14: 1893–1903. https://doi.org/10.2147/IDR.S299774.
127	(Rhouma et al. 2021)	Rhouma, Mohamed, Michelle Tessier, Cécile Aenishaenslin, Pascal Sanders, and Hélène Carabin. 2021. "Should the Increased Awareness of the One Health Approach Brought by the COVID-19 Pandemic Be Used to Further Tackle the Challenge of Antimicrobial Resistance?" *Antibiotics* 10 (4). https://doi.org/10.3390/antibiotics10040464.
128	(Zeitoun et al. 2021)	Frontline: A Call to Rethink War, WASH, and Public Health." *Annals of Global Health* 87 (1): 21. https://doi.org/10.5334/aogh.3140.
129	(Nag and Kaur 2021)	Nag, V.L., and N. Kaur. 2021. "Superinfections in COVID-19 Patients: Role of Antimicrobials." *Dubai Medical Journal* 4 (2): 117–26. https://doi.org/10.1159/000515067.
130	(Malcolm et al. 2020)	Malcolm, William, Ronald A Seaton, Gail Haddock, Linsey Baxter, Sarah Thirlwell, Polly Russell, Lesley Cooper, Anne Thomson, and Jacqueline Sneddon. 2020. "Impact of the COVID-19 Pandemic on Community Antibiotic Prescribing in Scotland." *JAC-Antimicrobial Resistance* 2 (4): dlaa105. https://doi.org/10.1093/jacamr/dlaa105.
